# Evaluation of the 5-HT_2C_ receptor drugs RO 60-0175, WAY 161503 and mirtazepine in a preclinical model of comorbidity of depression and cocaine addiction

**DOI:** 10.1007/s43440-022-00428-2

**Published:** 2022-11-14

**Authors:** Joanna Jastrzębska, Małgorzata Frankowska, Irena Smaga, Magdalena Hubalewska-Mazgaj, Agata Suder, Renata Pieniążek, Edmund Przegaliński, Małgorzata Filip

**Affiliations:** Maj Institute of Pharmacology, Department of Drug Addiction Pharmacology, Polish Academy of Sciences, Smętna 12, 31-343 Kraków, Poland

**Keywords:** 5-HT_2C_ receptor, Addiction, Depression, Cocaine self-administration, Bulbectomy, Rats

## Abstract

**Background:**

Epidemiological data indicate a high rate of comorbidity of depression and cocaine use disorder (CUD). The role of serotonin 2C (5-HT_2C_) receptors in the mechanisms responsible for the coexistence of depression and CUD was not investigated.

**Methods:**

We combined bilateral olfactory bulbectomy (OBX), an animal model of depression, with intravenous cocaine self-administration and extinction/reinstatement in male rats to investigate two 5-HT_2C_ receptor agonists (Ro 60-0175 (RO) and WAY 161503 (WAY)) and the 5-HT_2C_-receptor preferring antagonist mirtazapine (MIR; an antidepressant), with the goal of determining whether these drugs alter cocaine-induced reinforcement and seeking behaviors. Additionally, neurochemical analyses were performed following cocaine self-administration and its abstinence period in the brain structures in OBX rats and SHAM-operated controls.

**Results:**

Acute administration of RO reduced, while WAY non-significantly attenuated cocaine reinforcement in both rat phenotypes. Moreover, RO or WAY protected against cocaine-seeking behavior after acute or after repeated drug administration during extinction training in OBX and SHAM rats. By contrast, acutely administered MIR did not alter cocaine reinforcement in both rat phenotypes, while it’s acute (but not repeated) pretreatment reduced cocaine-seeking in OBX and SHAM rats. In neurochemical analyses, cocaine reinforcement increased 5-HT_2C_ receptor levels in the ventral hippocampus; a preexisting depression-like phenotype enhanced this effect. The 10-daily cocaine abstinence reduced 5-HT_2C_ receptor expression in the dorsolateral striatum, while the coexistence of depression and CUD enhanced local receptor expression.

**Conclusion:**

The results support a key role of 5-HT_2C_ receptors for treating CUD and comorbid depression and CUD. They may be backs the further research of pharmacological strategies with drug targeting receptors.

**Graphical abstract:**

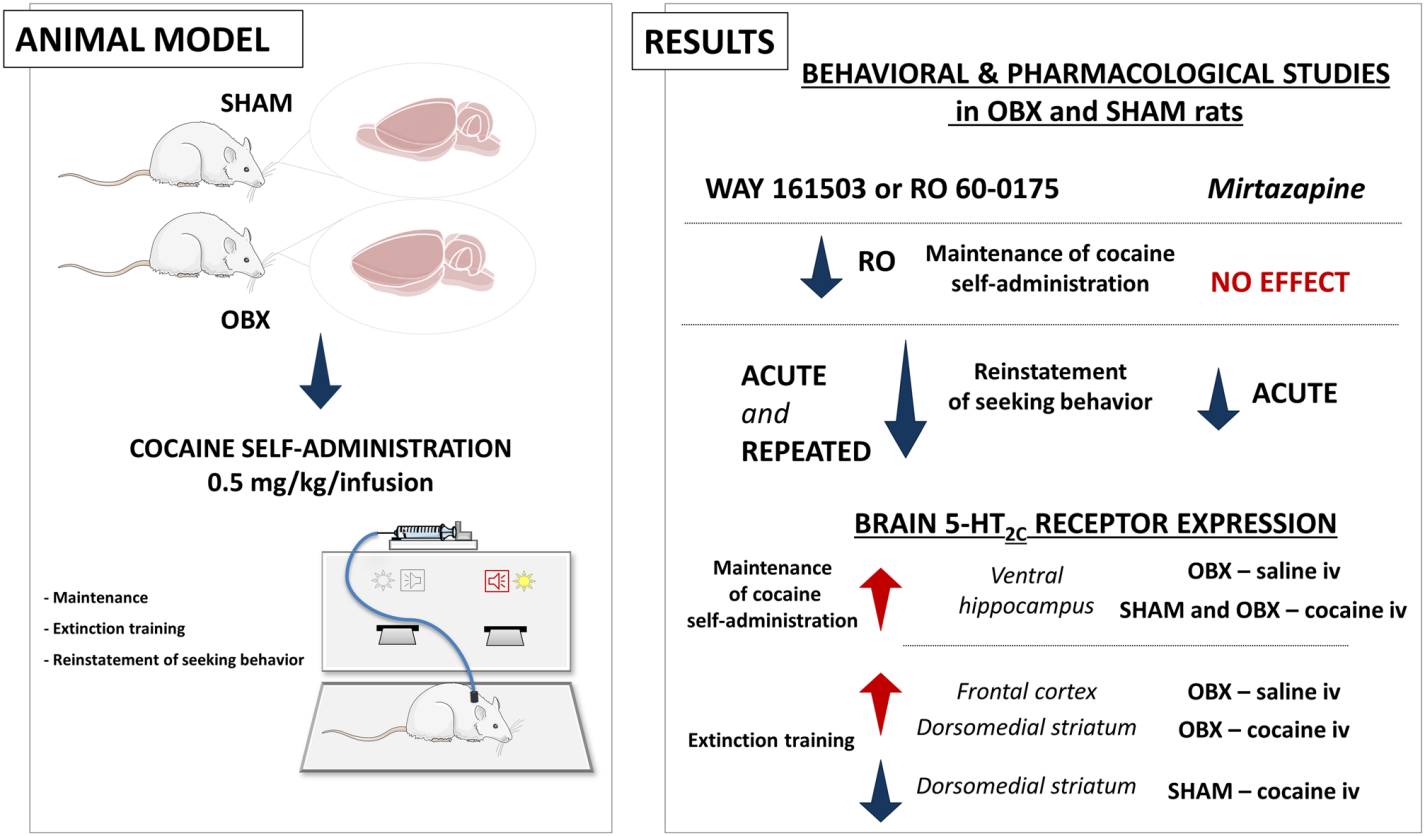

**Supplementary Information:**

The online version contains supplementary material available at 10.1007/s43440-022-00428-2.

## Introduction

Depressive disorders represent one of the most common mental disorders, representing 3.8% of the world population, and are associated with deterioration of social functioning and the quality of life [1]. Epidemiological and clinical data indicate a high rate of comorbidity of depression and substance abuse. One-third of patients suffering from depression have been diagnosed with a substance use disorder [2, 3]. The frequency of the coexistence of these disorders depends on the type of substance of abuse: 54% of patients with depression abuse opioids, 38% alcohol, and 32% psychostimulants [4]. In contrast, research conducted by Rounsaville et al. [5] relating only to cocaine use disorder (CUD) showed a very high occurrence (60%) of this disorder among people suffering from depression. The primary depressive disorder leads to the use of psychostimulants (e.g., cocaine) to self-medicate the symptoms of depression. Khantzian first described the ‘self-medication’ hypothesis [6, 7]. According to its assumptions, patients with depressive disorders take cocaine to relieve symptoms of depression, and hypomania or hyperactivity [6, 7].

Based on several preclinical and clinical observations, in both disorders, serotonin (5-HT) receptors (mainly 5-HT_2C_ subtypes) play a crucial role [8]. Human and animal research indicates that these receptors are implicated in depression. Thus, the clinically approved atypical antidepressants mirtazapine (MIR) and mianserin [9] as well as agomelatine (with additional melatonin 1 and 2 receptor agonism properties); [10–12] have clear antagonistic 5-HT_2C_ receptor profiles [11, 13–16]. Prolonged treatment with various antidepressants leads to adaptation with a reduction in the number of active 5-HT_2C_ receptors in the central nervous system [17–19]. On the other hand, both 5-HT_2C_ receptor agonists and antagonists showed antidepressant properties in animal models of depression [20–22]; see [11, 23–27] and desensitization of the 5-HT_2C_ in 5-HT receptor transporter knockout mice shows antidepressant-like effects [28, 29].

Considering CUD, 5-HT_2C_ receptors are valid therapeutic targets for treating various addictive processes. Since the early years of the twenty-first century, several laboratories reported inhibitory effects of selective 5-HT_2C_ receptor stimulation toward brain stimulation reward voluntary cocaine intake as well as reinforcing and cue reactivity in rodents and non-human primates [30–42]. In line with preclinical observations, abstinent cocaine users exhibit lower sensitivity to the effects of 5-HT_2C_ receptor agonists [43–46]. Using genetic and pharmacological tools, 5-HT_2C_ receptor blockade produces opposite effects on cocaine reinforcement and the reinstatement of drug-seeking in rodents [35, 47, 48] or in primates ([32, 33]; see also [34]).

Epidemiological and clinical data indicate a high rate of comorbidity of depression and substance abuse. As described above, the role of 5-HT_2C_ receptors in the mechanisms responsible for CUD or depression is well-established. However the role of this receptor has not been investigated in the coexistence of both disorders. To model the depressive phenotype, we chose bilateral olfactory bulbectomy (OBX). It is one of the widely used rat models of depression [49], which exhibits behavioral, neuroanatomical, neurochemical, immune, and endocrine changes that closely mimic the syndromes observed in patients suffering from major depression [49, 50]. Here, the OBX model was combined with the rodent cocaine self-administration procedure which is well-recognized as a translational model with predictive validity for human drug-taking, and critically important to understanding the molecular and physiological processes underlying CUD [51]. Our previous research demonstrates that the occurrence of a depressive-like phenotype before introduction to cocaine self-administration enhances subsequent drug abstinence with extinction training and cocaine-seeking behaviors; however, it does not significantly influence cocaine's rewarding properties [52–54]. To clarify the role of 5-HT_2C_ receptors and to evaluate the efficacy of several 5-HT_2C_ receptor drugs, including the one-side receptor agonists RO 60–0175 (RO) and WAY 161503 (WAY) and on the other side used antidepressant MIR. MIR has a dual action as a mixed monoamine agonist–antagonist, facilitating norepinephrine, 5-HT, and dopamine release in mesocorticolimbic areas involved in drug reward, craving, and seeking [55, 56]. MIR has historically been considered an antagonist at 5-HT_2C_ and other receptors [25]; however, more recent studies indicate its action as an inverse agonist at constitutively active 5-HT_2C_ receptors [57]. Additionally, to clarify the results obtained for 5-HT_2C_ receptor drugs, we performed neurochemical analyses following cocaine self-administration and its abstinence period in the brain structures linked to CUD and depression in OBX rats and SHAM controls.

## Materials and methods

### Animals

Male Wistar rats (Charles River, Germany; initial weight 280–300 g) were used. The animals were housed up to 5/cage (training procedures) or individually (self-administration procedures) in standard rodent cages at a room temperature of 22 ± 2 °C and 45–65% humidity with a 12-h light–dark cycle (lights on at 6:00). The animals had free access to food (Labofeed pellets) and water during a 7-day habituation period. Following habituation, the rats were given limited access to water (no longer than 6 h/day) during the initial lever-press training sessions. All experiments were conducted during the light phase of the light–dark cycle (between 07:00 and 16:00). The initial experimental groups consisted of 8–14 rats each. All experiments were carried out by the European directive 2010/63/EU on the protection of animals used for scientific purposes and were approved by the Animal Care and Use Committee of the Institute of Pharmacology, Polish Academy of Sciences in Krakow (no. 1261/2015).

### Drugs

Cocaine hydrochloride (Sigma-Aldrich, St. Louis, USA) were dissolved in sterile 0.9% NaCl and administered *iv* in a volume of 0.1 ml/infusion or *ip* in a volume of 1 ml/kg directly before the sessions. Mirtazapine (MIR, Kemprotec, UK), RO 60-0175 (RO, Tocris, Germany), and WAY 161503 (WAY, Tocris, Germany) were dissolved in sterile 0.9% NaCl, and given *ip* at 15 min (WAY), 20 min (RO) and 30 min (MIR) before test sessions in a volume of 1 ml/kg.

### Behavioral procedures

**Fig. 1:**
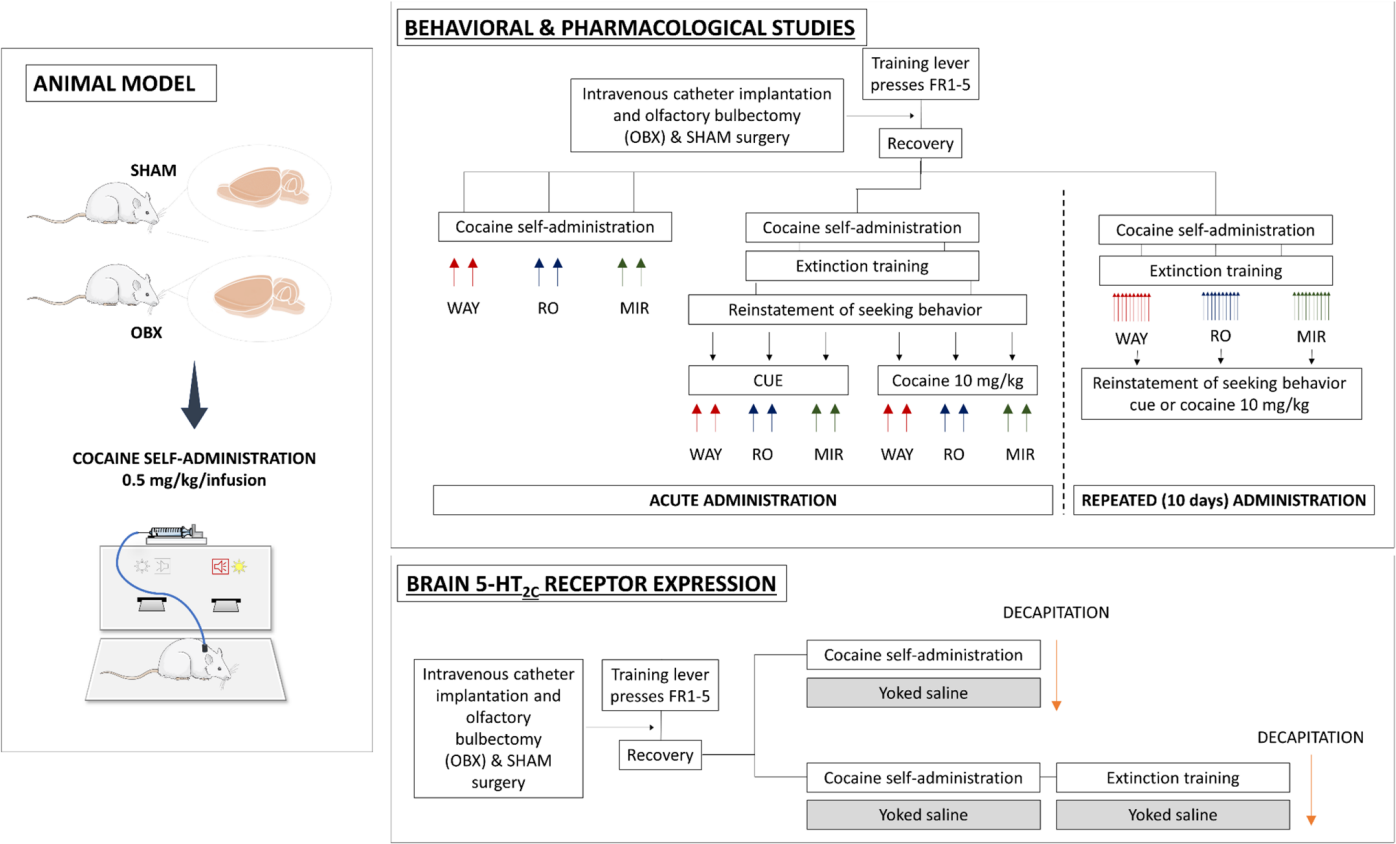
Experimental design for behavioral and pharmacological (with using 5-HT_2C_ receptor drugs WAY 161503 (WAY), RO 60-0175 (RO) and mirtazapine (MIR)) as well as neurochemical (5-HT_2C_ protein expression) analyses. Behavior and 5-HT_2C_ receptor expression—each line represents a separate group of rats. Arrows represent exposure to a reinstatement stimulus (black) or administration of drugs (red, blue, green) or decapitation (orange)

Behavioral procedures of lever training, catheter implantation, olfactory bulbectomy, yoked procedure and maintenance of cocaine self-administration, extinction training, and the reinstatement of the cocaine-seeking behavior were performed according to the procedures devised by Frankowska et al. [52] and described in detail previously by Jastrzębska et al. [53, 54]. As shown in Fig. 1, rats were first trained to lever-press, in 2-h daily sessions under the fixed ratio (FR) schedule 1 of water reinforcement; the FR increased to FR5 during the next days. Two days after the training time under FR5 (food and water ad libitum), the rats were anesthetized with ketamine HCl (75 mg/kg, *ip*, Bioketan, Biowet, Poland) and xylazine (5 mg/kg, *ip*, Sedazin, Biowet, Poland). Later, they were chronically implanted with a catheter in the external jugular vein. During post-surgery recovery, the catheters were flushed daily with 0.1 ml of a heparinized saline solution (70 U/ml, Biochemie, Austria) or 0.1 ml of a cephazolin solution (10 mg/ml Biochemie GmbH, Austria).

#### Olfactory bulbectomy

The removal of the olfactory bulbs or SHAM surgery was carried out in parallel with the catheter implantation. The procedure has been described previously by Jastrzębska et al. [53, 54] A midline incision was made from approximately 1 cm posterior to 1 cm anterior to the bregma. Burr holes (2 mm diameter) were then carefully drilled through the skull, approximately 7 mm anterior to the bregma and 2 mm on either side of the midline. The olfactory bulbs were then gently removed by suction and care was taken to avoid damage to the cortex. The burr holes were filled with hemostatic sponges and the wound was closed with surgical thread under aseptic conditions. SHAM controls were treated in a similar manner, with the exception of the removal of the olfactory bulbs. The OBX an SHAM rats were given an analgesic for 5 days; meloxicam (0.2 mg/ml; vol. 0.2 ml/rats, *sc*; Boehringer Ingelheim, Germany). During recovery, the rats were handled every day to eliminate any aggressiveness that would otherwise develop [50, 58]. Verification of the correctness of bulbectomy was carried out at the end of behavioral experiments. The reconvalescence of animals lasted 14 days before experiments began.

#### Maintenance of cocaine self-administration, extinction training, and reinstatement of cocaine-seeking

Following the recovery, OBX and SHAM groups were retrained to press the lever associated with water reinforcement, and on the next day, the animals were trained to self-administer cocaine at a dose of 0.5 mg/kg/infusion with an FR5 schedule of reinforcement. The reason we chose 0.5 mg/kg/infusion of cocaine to evaluate the effect of various 5-HT_2C_ receptor’ agents was that this dose of cocaine in Wistar rats evoked a comparable or higher intake compared to doses of lower 0.25 mg/kg/infusion or higher 1 mg/kg/infusion and produced a faster and greater increase in the rate of self-administration than a dose above 1 mg/kg/infusion as shown previously in our and other previous studies of the dose–effect curve for cocaine self-administration [53, 54, 59, 60]. Each completion of the schedule by pressing the active lever resulted in a 5-s injection of cocaine and a 5-s presentation of the stimulus complex that consisted of the activation of the white stimulus light directly above the active lever and a tone from the generator (2000 Hz; 15 dB above the ambient noise level). Twenty seconds after each injection (time-out period), the next injection was impossible. During this time the responses were recorded, but there were no programmed consequences. Pressing the inactive lever was recorded, but never resulted in cocaine delivery.

Separate groups of cocaine self-administered rats that met the maintenance criterion (active lever presses during 3 consecutive days varied by 15% or less, and a minimum of 10 infusions per session) were used in the extinction and reinstatement tests induced by either a non-contingent priming injection (10 mg/kg cocaine, *ip*) or a conditioned cue (the tone + light) being previously paired with cocaine reinforcement. Active lever presses during the reinstatement tests resulted in an intravenous injection of saline only.

To analyze the expression of 5-HT_2C_ receptors, the yoked saline procedure was introduced. In that procedure, each rat actively self-administering cocaine (or saline during the extinction phase) has been assigned to rat that was passively receiving *iv* saline in the same amount and manner as the active animal. Two cohorts of OBX and SHAM rats (6 rats/group) were sacrificed immediately following the last cocaine self-administration session or after the last extinction session.

In pharmacological studies, OBX and SHAM rats (6–9 rats/group) were pretreated with acute injections of 5-HT_2C_ receptor drugs (WAY: 1–3 mg/kg, RO: 3–10 mg/kg, MIR: 5–20 mg/kg,) or corresponding vehicles before cocaine self-administration sessions. The test sessions were separated by at least two to three baseline days of cocaine self-administration.

In further analyses, to study the effects of 5-HT_2C_ receptor drugs on cocaine-seeking behavior, the other cohorts of OBX and SHAM rats (6–10 rats/group) were acutely pretreated with MIR (2.5–20 mg/kg), WAY (0.1–1 mg/kg), RO (0.3–3 mg/kg) or the corresponding vehicles before the reinstatement test sessions. Each rat underwent only one type of reinstatement procedure, in which either cocaine (10 mg/kg, *ip*) or the cue was presented in a maximum of 4 tests. The order of drug injections was balanced according to a Latin square design, and the drug combinations were given in a randomized order. The test sessions were separated by at least two to three extinction sessions.

Additionally, separate groups of OBX and SHAM rats (6–11 rats/groups) were repeatedly (1 daily injection over 10 days) pretreated with WAY (1 mg/kg), RO (1 mg/kg), MIR (10 mg/kg) or the corresponding vehicles during extinction phase and then cocaine or cue-induced reinstatement tests were performed. The test sessions were separated by at least two to three extinction sessions. The doses of WAY, RO and MIR were selected based on the acute drug effects.

### Western blot

Brain structures isolated immediately after behavioral procedures were homogenized in a homogenization buffer (1 mM HEPES, 0.1 M DTT, 0.1 mM EGTA (pH 7.2–7.8), COMPLETE and sterile water) using a homogenizer ball (Bioprep-24, Allsheng, China) at 10 s at 10,000 rpm, and were then denatured for 2 min at 85 °C, 2 min in ice, 5 min at 85 °C, and finally 2 min in ice as described previously by Smaga et al. [61]. The bicinchoninic acid assay protein assay kit (Serva, Germany) was used for protein determination. Protein samples (40 mg) were resolved in gels and transferred to membrane, and determination of 5-HT_2C_ receptor was performed using anti-5-HT_2C_ rabbit monoclonal antibody (1:1000; Abcam, UK). The expressions of 5-HT_2C_ receptors were evaluated relative to anti-glyceraldehyde 3-phosphate dehydrogenase antibody (GAPDH), using rabbit anti-GAPDH polyclonal antibody (1:1500, sc-25778, Santa Cruz Biotechnology, USA). Blots were washed and incubated with goat anti-rabbit secondary antibody (1:6000; 926–68,071; Li-cor, USA) and visualized with fluorescence detection Odyssey Clx (Licor, USA). The analysis was performed using Image Studio v.2.1. All data were expressed as % of control.

### Statistical analysis

Statistical analyses for data from the behavioral experiments were performed using mixed ANOVA for the following factors under the specific protocols: (i) cocaine self-administration with acute drug treatment: surgical condition (SHAM and OBX), pretreatment (vehicle and drug), lever (active and inactive), and their interaction; or surgical condition (SHAM and OBX), pretreatment (vehicle and drug), and their interaction; (ii) extinction with repeated drug treatment: surgical condition (SHAM and OBX), session (from 1 to 9), pretreatment (vehicle and drug), and their interaction; iii) reinstatement tests with acute drug treatment: surgical condition (SHAM and OBX), lever (active and inactive), reinstatement (cue or cocaine), and their interaction; or surgical condition (SHAM and OBX), pretreatment (vehicle and drug), lever (active and inactive), and their interaction; iv) reinstatement following repeated drug treatment during extinction: surgical condition (SHAM and OBX), pretreatment (vehicle and drug), lever (active and inactive), and reinstatement (extinction and cue or cocaine), and their interaction. Post hoc Newman-Keuls test was used to analyze the differences between the group means. Animals that did not complete the self-administration acquisition/maintenance (2–6 rats in SHAM or OBX group), extinction criteria (1–2 rats in OBX group), or did not maintain extinction criteria between tests (1–2 rats in OBX group) were excluded from the behavioral tests and the data analysis.

Data from the Western blot were analyzed using the GraphPad PRISM 5.0 (GraphPad Software, San Diego, USA). Statistical analyses covered (i) two-way analysis of variance (ANOVA) with surgical condition and treatment as factors or (ii) Student’s *t* test. Group differences after significant ANOVAs were analyzed by the post hoc Newman–Keuls test.

The criterion for a statistically significant difference was set at *p* < 0.05.

## Results

### Behavioral experiments

#### Maintenance of cocaine self-administration

**Fig. 2:**
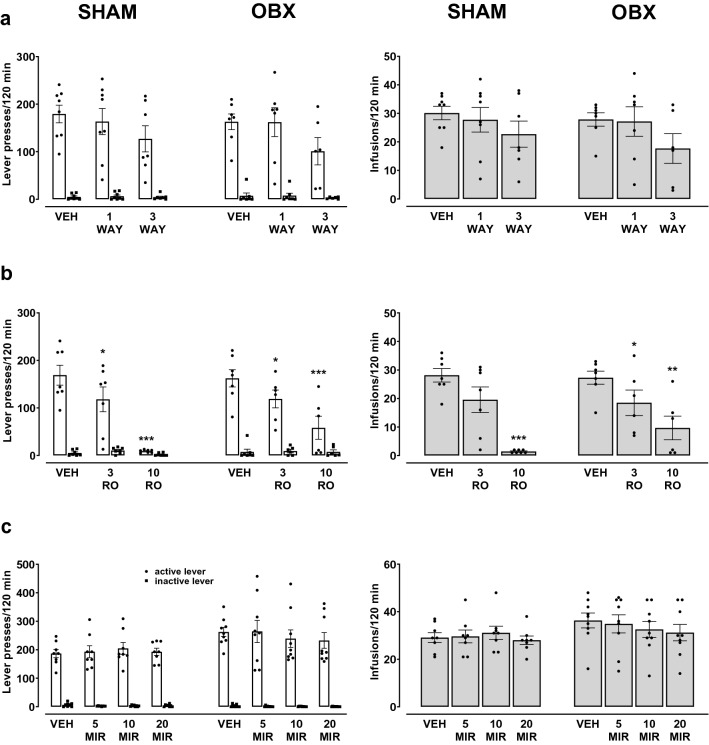
Effects of acute WAY 161503 (WAY; 1 and 3 mg/kg, *ip*, **a**), RO 60-0175 (RO; 3 and 10 mg/kg, *ip*, **b**) or mirtazapine (MIR, 5–20 mg/kg, *ip*, **c**) treatment on the maintenance of cocaine (0.5 mg/kg/infusion) self-administration under the FR5 schedule of reinforcement in the bulbectomized (OBX) and SHAM-operated rats. The number of active and inactive lever presses as well as cocaine infusions are expressed as the means (± SEM) of the data from 6 to 9 rats/group. The statistical analyses were done with one or two-way ANOVA with post hoc Newman–Keuls test. *p < 0.05, **p < 0.01, ***p < 0.001 versus vehicle (VEH)

Figure 2a shows the effects of the acute WAY administration in OBX and SHAM rats on the active and inactive lever presses, and on the number of cocaine infusions. A three-way ANOVA did not show differences between the groups treated with WAY (the surgical condition × pretreatment × lever: *F*_2,74_ = 0.10, *p* = 0.91), and the analysis inside each experimental group treated with WAY did not indicate a difference in the cocaine reinforcement (a two-way ANOVA: *F*_2,34_ = 1.50, *p* = 0.24 and *F*_2,40_ = 1.10, *p* = 0.34, respectively).

Acute treatment with WAY did not alter the number of cocaine infusions in both animal groups, as shown by a two-way ANOVA for the pretreatment × lever interaction in OBX (*F*_2,17_ = 1.60, *p* = 0.23) and SHAM groups (*F*_2,20_ = 0.93, *p* = 0.40); the observed effect was independent of surgical manipulation (*F*_3,44_ = 0.24, *p* = 0.86).

The number of active and inactive lever presses and the number of cocaine infusions for OBX and SHAM animals after RO administration are shown in Fig. 2b. A three-way ANOVA for the surgical condition × pretreatment × lever interaction did not show a difference between the types following RO treatment (*F*_2,66_ = 0.48, *p* = 0.62), which means that the administration of RO had a similar effect on the number of presses on the active lever in both groups of animals. Pretreatment with RO in a dose-dependent manner decreased the number of presses of the active, but not the inactive lever in both groups of rats. Individual two-way ANOVA analyses for OBX and SHAM groups indicated a significant effect for the pretreatment × lever interaction (*F*_2,30_ = 5.54, *p* < 0.01 and *F*_2,36_ = 16.66, *p* < 0.001, respectively).

Removal of the olfactory bulbs did not affect the number of cocaine infusions after acute administration of RO (*F*_2,33_ = 1.21, *p* = 0.34). A one-way ANOVA for each group showed a significant effect of RO (3–10 mg/kg) on the cocaine reinforcement in OBX (*F*_2,15_ = 7.54, *p* < 0.01) and SHAM rats (*F*_2,18_ = 21.67, *p* < 0.001), but only at a dose of 10 mg/kg (Fig. 2b).

The lack of significant effects after acute administration of MIR in OBX and SHAM rats was demonstrated with a three-way ANOVA (*F*_2,182_ = 0.69, *p* = 0.57; Fig. 2c). The inner analysis for each experimental group treated with MIR did not indicate a difference in the cocaine reinforcement. MIR (5–20 mg/kg) did not alter the number of lever responses in OBX or SHAM rats (a two-way ANOVA: *F*_3,88_ = 2.30, *p* = 0.08 and *F*_3,94_ = 0.14, *p* = 0.92, respectively).

Acute treatment with MIR did not alter the number of cocaine infusions in both animal groups, as shown by a two-way ANOVA for the pretreatment × lever interaction in OBX (*F*_3,47_ = 0.37, *p* = 0.77) and SHAM groups (*F*_3,44_ = 1.60, *p* = 0.33). The observed response was independent of surgical manipulation (*F*_3,91_ = 0.89, *p* = 0.45).

#### Extinction training

**Fig. 3:**
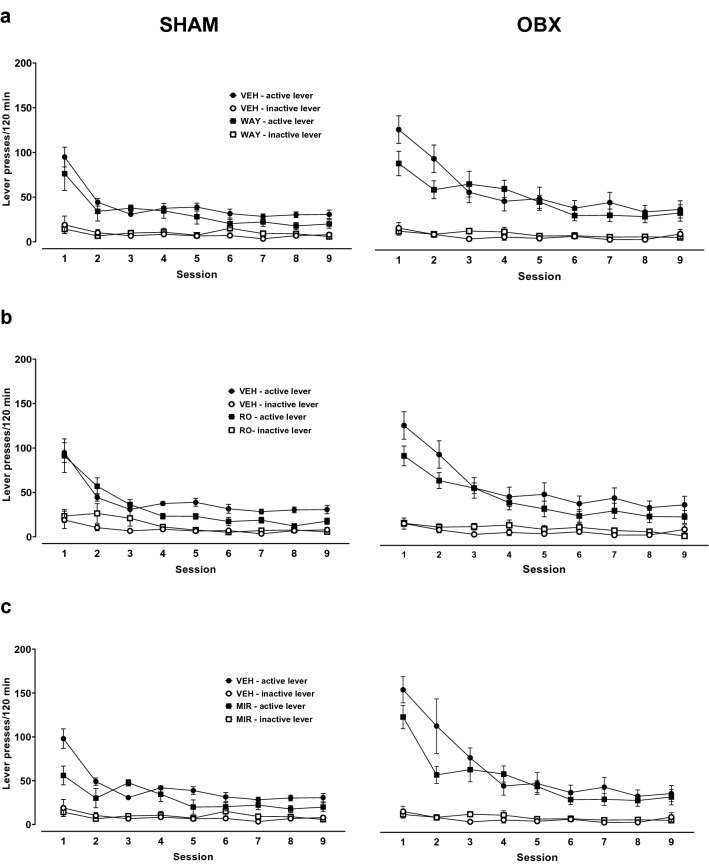
Effects of repeated treatment with WAY 161503 (WAY; 1 mg/kg, *ip*, **a**), RO 60-0175 (RO; 1 mg/kg, *ip*, **b**) and mirtazapine (MIR, 10 mg/kg, *ip*, **c**) or the corresponding vehicle (VEH) injected daily during the extinction training in the bulbectomized (OBX) and SHAM-operated rats. The number of active and inactive lever presses is expressed as the group means (± SEM) from 6 to 10 rats/group. The statistical analyses were done with multi-way repeated measures ANOVA

After cocaine self-administration, extinction training was introduced to all animals. Figure 3 shows the effects of the repeated administration of WAY, RO, or MIR, in OBX and SHAM rats on the active and inactive lever presses during extinction training.

A mixed repeated measures ANOVA did not show a significant effect of the repeated administration of WAY (1 mg/kg) or vehicle during extinction training in OBX and SHAM rats (the surgical condition × session × pretreatment × lever interaction (*F*_8,464_ = 0.68, *p* = 0.71). Similarly, a three-way repeated measures ANOVA for each group separately did not indicate a significant effect for the session × pretreatment × lever interaction in OBX (*F*_8,256_ = 1.30, *p* = 0.24) and SHAM rats (*F*_8,208_ = 3.40, *p* = 0.92; Fig. 3a).

Repeated administration of RO (1 mg/kg) during extinction training in SHAM and OBX rats did not cause changes in the number of lever presses (*F*_8,256_ = 0.52, *p* = 0.84 and *F*_8,208_ = 0.19, *p* = 0.99, respectively). Moreover, the effect of the session × pretreatment × lever interaction was the same in both groups of animals (*F*_8,454_ = 0.48, *p* = 0.87; Fig. 3b).

In like manner, the repeated administration of MIR (10 mg/kg) or vehicle did not significantly vary lever responses between experimental groups during the extinction training (*F*_8,608_ = 0.74, *p* = 0.65). The inner three-way repeated measures ANOVA for each group did not indicate a significant effect for the session × pretreatment × lever interaction in OBX (*F*_8,272_ = 1.18, *p* = 0.31) and SHAM rats (*F*_8,304_ = 0.99, *p* = 0.44; Fig. 3c).

#### Cue-induced reinstatement of seeking behavior: acute administration with 5-HT_2C_ receptor drugs

**Fig. 4:**
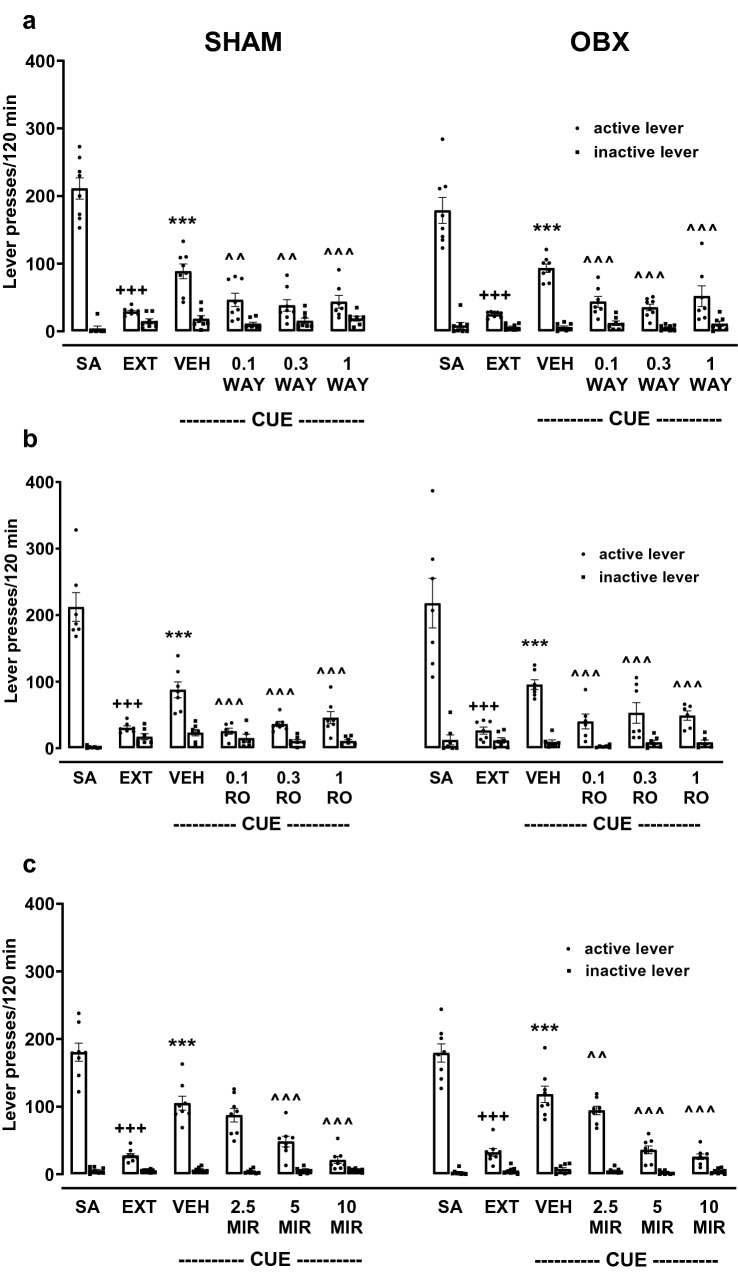
Effects of acute WAY 161503 (WAY; 0.1–1 mg/kg, *ip*, **a**), RO 60-0175 (RO; 0.1–1 mg/kg, *ip*, **b**) or mirtazapine (MIR, 2.5–10 mg/kg, *ip*, **c**) treatment on the reinstatement of the cocaine-seeking behavior induced by the cue (CUE; tone + light) in the bulbectomized (OBX, right panels) and SHAM-operated rats (left panels). The number of active and inactive lever presses is expressed as the means (± SEM) of the data from 6 to 10 rats/group. The statistical analyses were done with two-way ANOVA followed by post hoc Newman–Keuls test ***p < 0.001 versus extinction (EXT); ^^^*p* < 0.001 versus vehicle (VEH); ^+++^*p* < 0.001 versus cocaine self-administration (SA)

The results for the WAY experiment following the presentation of the cocaine-associated cue (tone + light) to OBX and SHAM groups were presented in Fig. 4a. The surgical condition did not cause changes in the number of lever presses after 10-days extinction training between groups (a three-way ANOVA: *F*_1,54_ = 1.01, *p* = 0.31). A two-way ANOVA in each group for the self-administration procedure × lever interaction showed a decrease in the number of active lever presses after extinction training in SHAM and OBX groups, as compared to cocaine self-administration (*F*_1,26_ = 193.02, *p* < 0.001 and *F*_1,28_ = 66.56, *p* < 0.001, respectively).

The surgical condition did not reveal an effect on behavioral responses during reinstatement seeking behavior induced by cue in both groups (*F*_1,54_ = 0.63, *p* = 0.43). A significant main effect for the reinstatement (cue) × lever interaction was observed in OBX and SHAM rats, and the significal increase (p < 0.001) in the number of active lever presses during the reinstatement of the drug-seeking behavior in rats was demonstrated (SHAM: *F*_1,26_ = 26.02, *p* < 0.001; OBX *F*_1,28_ = 36.28, *p* < 0.001).

A three-way ANOVA did not reveal an effect for the surgical condition × pretreatment × lever interaction (*F*_3,104_ = 0.42, *p* = 0.74), which indicates that there was no significant difference between OBX and SHAM rats in the effects of WAY. An individual two-way ANOVA for SHAM and OBX rats showed that pretreatment with WAY (0.1–1 mg/kg) significantly decreased the cue-induced reinstatement in SHAM (pretreatment × lever interaction: *F*_3,54_ = 3.04, *p* < 0.05) and OBX (*F*_3,50_ = 5.90, *p* < 0.05) animals, what proves that pretreatment with WAY effectively altered the reinstatement of the drug-seeking behavior (Fig. 4a).

For the RO experiment, the results for the cue-induced reinstatement of cocaine-seeking in OBX and SHAM animals were presented in Fig. 4b. A two-way ANOVA showed a significant reduction in active lever presses after 10 days of extinction training under both surgical conditions (OBX: *F*_1,24_ = 37.67, *p* < 0.001; SHAM: *F*_1,24_ = 74.61, *p* < 0.001). A two-way ANOVA revealed a significant effect for the reinstatement (cue) × lever interaction in both SHAM (*F*_1,24_ = 13.60, *p* < 0.01) and OBX (*F*_1,24_ = 39.89, *p* < 0.001) groups. The effect was independent of the surgical condition (*F*_1,46_ = 1.21, *p* = 0.27).

A three-way ANOVA (the surgical condition × pretreatment × lever interaction) did not show an effect for acute pretreatment with RO (0.1–1 mg/kg; *F*_3,90_ = 0.37, *p* = 0.76). An individual analysis with a two-way ANOVA in OBX or SHAM rats showed that pretreatment with RO significantly decreased the cue-induced reinstatement in OBX (pretreatment × lever: *F*_3,44_ = 4.15, *p* < 0.01) and SHAM (pretreatment × lever: *F*_3,46_ = 5.90, *p* < 0.01) animals, what indicates that pretreatment with RO effectively changed the reinstatement of the drug-seeking behavior (Fig. 4b).

The effects of the MIR experiment, following the presentation of the cocaine-associated cue, are shown in Fig. 4c. A two-way ANOVA revealed a significant effect for the self-administration procedure × lever interaction in both OBX (*F*_1,28_ = 24.93, *p* < 0.001) and SHAM (*F*_1,28_ = 15.34, *p* < 0.001) rats; a reduction in active lever presses after 10-days of extinction was observed in both phenotypes of rats. Exposure to the cue induced an increase (*p* < 0.001) in the number of active lever presses during the reinstatement of the drug-seeking behavior in OBX (a two-way ANOVA for reinstatement (cue) × lever interaction: *F*_1,28_ = 28.51, *p* < 0.001) and SHAM rats (*F*_1,28_ = 16.18, *p* < 0.001); the observed effect was independent of the surgical condition (*F*_1,68_ = 0.22, *p* = 0.64).

During cue-induced reinstatement, a significant reduction in the drug-seeking behavior in OBX and SHAM rats was observed, following acute administration of MIR (a two-way ANOVA: *F*_3,56_ = 24.37, *p* < 0.001 and *F*_3,56_ = 7.36, *p* < 0.001). Post hoc analyses showed a significant reduction in the drug-seeking behavior that occurred in both groups following administration of MIR at doses of 2.5–10 mg/kg in OBX rats and only for 5 and 10 mg/kg in SHAM rats. The effect of pretreatment with MIR on OBX and SHAM rats was regardless of the surgical condition (*F*_3,112_ = 1.33, *p* = 0.23).

#### Cocaine-induced reinstatement of seeking behavior: acute administration with 5-HT_2C_ receptor drugs

**Fig. 5:**
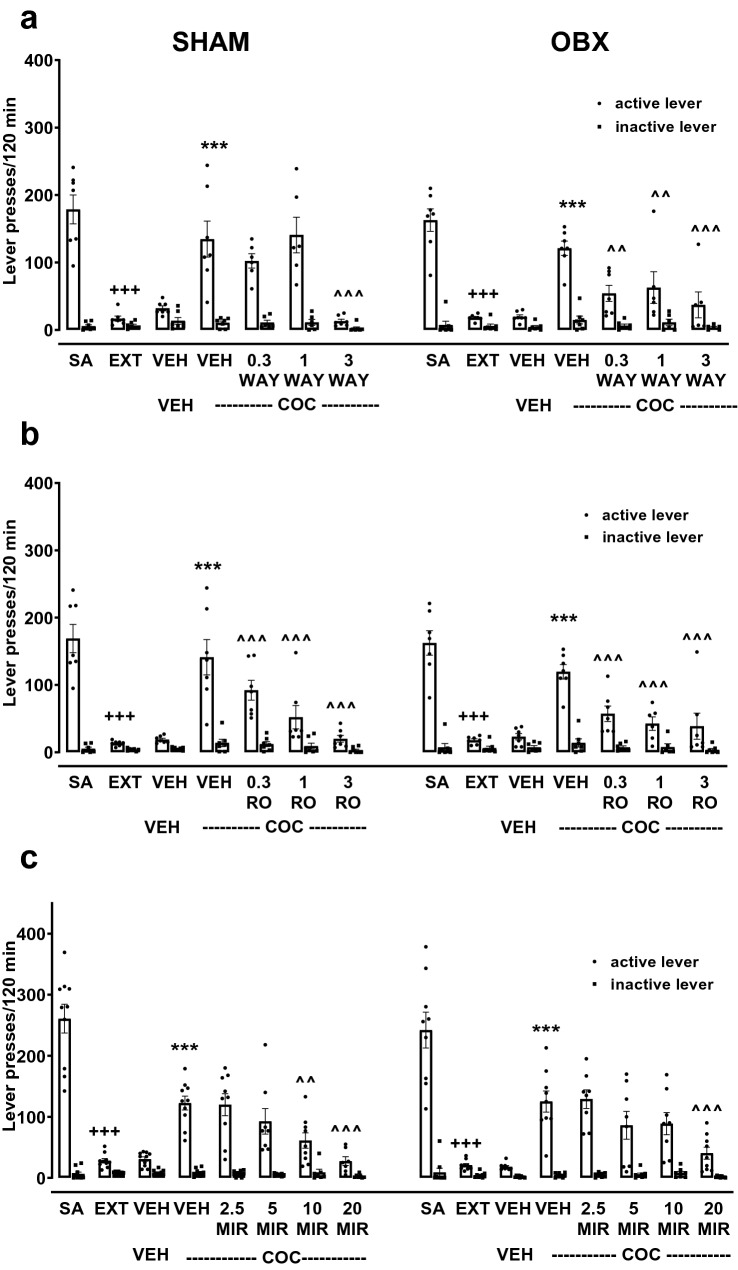
Effects of acute WAY 161503 (WAY; 0.3–3 mg/kg, *ip*, **a**), RO 60-0175 (RO; 0.3–3 mg/kg, *ip*, **b**) and mirtazapine (MIR, 2.5–20 mg/kg, *ip*, **c**) or the corresponding vehicle (VEH) treatment on the cocaine-induced (COC, 10 mg/kg, *ip*) reinstatement of cocaine-seeking behavior in the bulbectomized (OBX, right panels) and SHAM-operated rats (SHAM, left panels). The number of active and inactive lever presses is expressed as the means (± SEM) of the data from 6 to 10 rats/group. The statistical analyses were done with two-way ANOVA with post hoc Newman–Keuls test. ***p < 0.001 versus extinction (EXT); ^^*p* < 0.01, ^^^*p* < 0.01 versus VEH + COC; ^+++^*p* < 0.001 versus cocaine self-administration (SA)

Figure 5a shows the effects of acute WAY administration on cocaine-induced reinstatement in OBX and SHAM groups. A 10-day extinction training led to a reduction in active lever presses (*p* < 0.001), more than inactive lever presses, compared to the last cocaine self-administration session in both experimental groups (Fig. 5a; a two-way ANOVA for the self-administration procedure × lever interaction: OBX *F*_1,20_ = 39.68 *p* < 0.001; SHAM *F*_1,24_ = 17.45, *p* < 0.001). A reduction in active lever presses after 10 days of extinction was found for both phenotype.

A three-way ANOVA did not indicate an effect for the surgical condition × pretreatment × lever interaction (*F*_3,84_ = 2.32, *p* = 0.08), which indicates that there was no significant difference between OBX and SHAM rats in the effects of WAY. A two-way ANOVA demonstrated a significant effect for the pretreatment × lever interaction in SHAM and OBX rats (*F*_3,44_ = 8.02, *p* < 0.001 and *F*_3,40_ = 2.92, *p* < 0.05, respectively). Post hoc analyses showed a significant reduction in the drug-seeking behavior occurred in both groups following administration of WAY, for all doses (0.3–3 mg/kg) in OBX rats and only for the WAY dose of 3 mg/kg in SHAM rats.

For the RO experiment, a two-way ANOVA showed a significant reduction in the active lever presses after 10 days of extinction training for both types of surgery conditions (OBX: *F*_1,24_ = 37.68, *p* < 0.001; SHAM: *F*_1,24_ = 74.61, *p* < 0.001). The effect was independent of the surgical condition (*F*_1,48_ = 0.54, *p* = 0.46; Fig. 5b). Administration of cocaine at a dose of 10 mg/kg (*ip*) increased in the responses to the active lever in OBX (*F*_1,24_ = 53.98, *p* < 0.001) and SHAM groups (*F*_1,24_ = 19.26, *p* < 0.001). A three-way ANOVA did not indicate an effect for the surgical condition × pretreatment × lever interaction (*F*_3,93_ = 0.78, *p* = 0.51), which showed no significant difference between OBX and SHAM rats in the effects of RO. A two-way ANOVA analysis demonstrated a significant effect for the pretreatment x lever interaction in SHAM and OBX rats (*F*_3,48_ = 7.07, *p* < 0.001 or *F*_3,45_ = 7.29, *p* < 0.001, respectively; Fig. 5b).

For the acute MIR administration cocaine-induced reinstatement in OBX and SHAM groups were presented in Fig. 5c. A two-way ANOVA for the self-administration procedure × lever interaction showed a significant reduction in active lever presses after 10 days of extinction in both experimental groups (OBX *F*_1,32_ = 23.86, *p* < 0.001; SHAM *F*_1,36_ = 95.16, *p* < 0.001); the surgical condition did not cause changes in behavioral responses in any phenotype (*F*_1,68_ = 0.12, *p* = 0.73).

Regarding the cocaine-induced seeking behavior, measured as increases of active lever responses (*p* < 0.001), a two-way ANOVA revealed an effect for the cue-induced reinstatement of seeking behavior in both experimental groups (OBX: *F*_1,30_ = 31.37, *p* < 0.001 and SHAM: *F*_1,36_ = 20.00, *p* < 0.001).

When MIR (2.5–20 mg/kg) was administered before placing rats in the experimental cage, a marked reduction in the number of active lever responses induced by cocaine was observed (a two-way ANOVA: OBX *F*_4,74_ = 3.81, *p* < 0.001; SHAM *F*_4,78_ = 4.08, *p* < 0.01). A post hoc test demonstrated a reduction in active lever presses in rats treated with 10 and 20 mg/kg doses of MIR for OBX, and only MIR for 20 mg/kg in SHAM rats. A three-way ANOVA did not indicate an effect for the surgical condition × pretreatment × lever interaction (*F*_4,150_ = 0.14, *p* = 0.97), which demonstrated no significant difference between OBX and SHAM rats in the effects of MIR.

#### Cue-induced reinstatement of seeking behavior: repeated 5-HT_2C_ receptor drug administration during extinction training

**Fig. 6:**
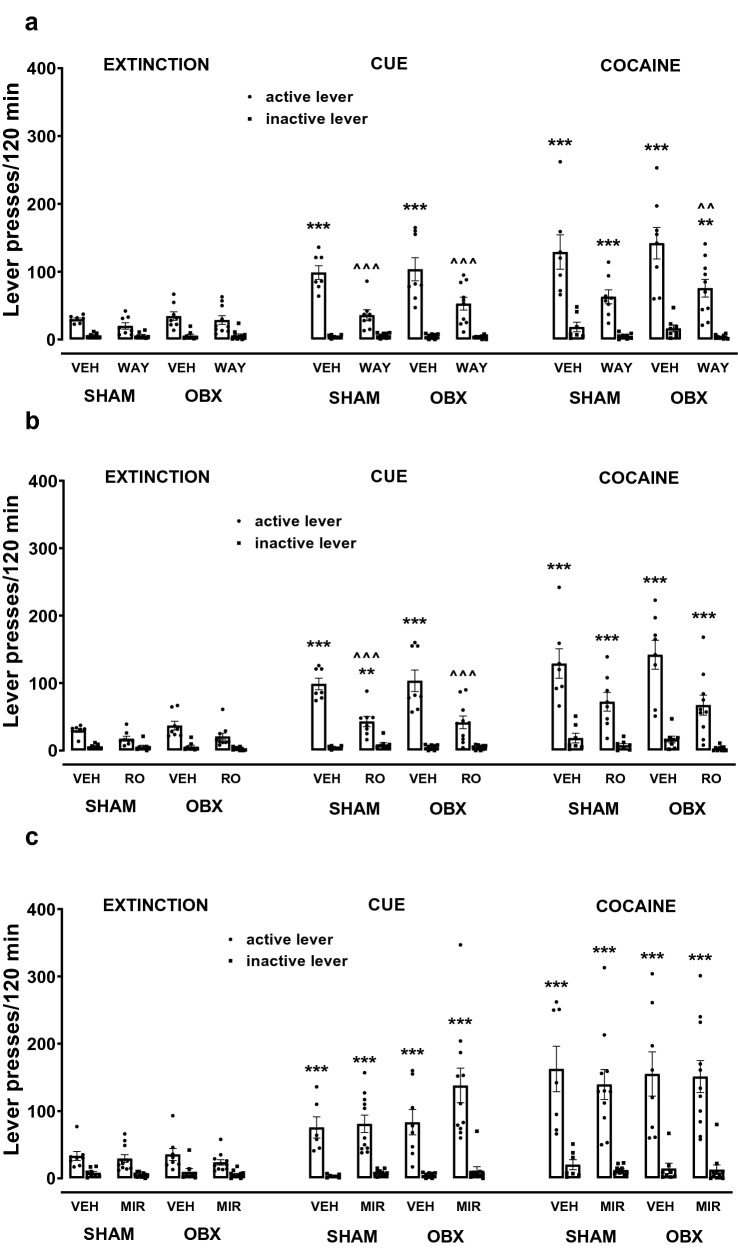
Effects of repeated WAY 161503 (WAY, 1 mg/kg, *ip*; **a**), RO 60-0175 (RO, 1 mg/kg, *ip*; **b**), mirtazapine (MIR, 10 mg/kg, *ip*; **c**) or corresponding vehicle (VEH) treatment during extinction training on the reinstatement of the cocaine-seeking behavior. MIR (10 mg/kg, *ip*), WAY (1 mg/kg, *ip*), RO (1 mg/kg, *ip*), or VEH were injected daily during the extinction training in the bulbectomized (OBX) and SHAM-operated control rats. The reinstatement of the cocaine-seeking behavior was initiated using the cue or cocaine (10 mg/kg, *ip*). The number of active and inactive lever presses is expressed as the group means (± SEM) from 6 to 11 rats/group. The statistical analyses were done with three-way ANOVA followed by post hoc Newman–Keuls test. **p < 0.01, ***p < 0.001 versus corresponding group during extinction; ^^*p* < 0.01, ^^^*p* < 0.001 versus corresponding group treated with VEH

After 10-daily administration of vehicle or WAY (1 mg/kg) during the extinction-training period, rats were tested for the response reinstatement induced by a cocaine-associated cue (Fig. 6a). As shown by a mixed ANOVA for the surgical condition × pretreatment × reinstatement (cue) × lever interaction, repeated WAY administration did not alter the cue-induced reinstatement for either type of surgery condition (*F*_1,116_ = 0.17, *p* = 0.68; Fig. 6a).

In the SHAM-operated animals, rats after repeated WAY administrations during extinction training reacted differently to cue-induced reinstatement (*F*_1,52_ = 15.09, *p* < 0.001). A post hoc analysis showed a significant increase in the number of active lever presses (*p* < 0.001) in the group receiving the vehicle, while in the group chronically treated with WAY, a significant reduction in the cue-induced relapse (*p* < 0.001) was observed, as compared to the vehicle group. In the OBX group, the differences were also observed in the response to cue-induced reinstatement between rats receiving repeated doses of WAY or vehicle (*F*_1,64_ = 14.24, *p* < 0.05; Fig. 6a). As demonstrated by the post hoc analysis, WAY administered chronically significantly attenuated the cue-induced relapse (*p* < 0.001; Fig. 6a).

In like manner, a mixed ANOVA for both types of surgery conditions did not show a significant effect of the surgical condition × pretreatment × reinstatement (cue) × lever interaction (*F*_1,116_ = 0.01, *p* = 0.94; Fig. 6b), which proves that pretreatment with RO (1 mg/kg) had the same effect for both surgery conditions. However, for these conditions, a three-way ANOVA showed a significant effect of the reinstatement (cue) × lever × pretreatment interaction in SHAM (*F*_1,52_ = 12.16, *p* < 0.001) and OBX (*F*_1,64_ = 5.49, *p* < 0.05) rats. A post hoc analysis showed a significant reduction in the cue-induced relapse for both surgery conditions (*p* < 0.001) after chronic administration of RO.

As shown by a mixed ANOVA for the surgical condition × pretreatment × reinstatement (cue) × lever interaction (*F*_1,145_ = 1.26, *p* = 0.26), the reinstatement of seeking behavior induced by a cue was relevant following either vehicle or MIR (10 mg/kg) pretreatment in both OBX or SHAM rats (Fig. 6c). Moreover, inner analyses for each group did not reveal a significant difference between treatment with vehicle or MIR, suggesting that rats responded similarly to the cue (OBX: *F*_1,68_ = 2.28, *p* = 0.14; SHAM: *F*_1,76_ = 0.25, *p* = 0.62).

#### Cocaine-induced reinstatement of seeking behavior: repeated drug administration with 5-HT_2C_ receptor drugs during extinction training

Following 10-days of vehicle or WAY administration during the extinction training SHAM and OBX rats were tested for the response reinstatement induced by cocaine (10 mg/kg, *ip*; Fig. 6a). A mixed ANOVA for both surgery conditions did not reveal a significant effect of repeated administration of WAY during extinction training for the pretreatment × surgical condition × cocaine × lever interaction (*F*_1,116_ = 0.02, *p* = 0.90), what indicates that the surgery condition did not correlate with a behavioral response to a priming cocaine injection (Fig. 6a). Similarly, a separate three-way ANOVA for SHAM (*F*_1,51_ = 2.31, *p* = 0.13) and OBX (*F*_1,64_ = 2.73, *p* = 0.10) rats showed no significant effect of WAY on the cocaine-induced reinstatement (Fig. 6a).

After 10 days of repeated treatment with the vehicle or RO, during the extinction training, SHAM and OBX rats were tested for the response reinstatement induced by cocaine (10 mg/kg, *ip*). For both types of surgery conditions, a mixed ANOVA did not reveal a significant effect for the pretreatment × surgical condition × cocaine × lever interaction (*F*_1,116_ = 0.14, *p* = 0.71), which provides evidence that OBX and SHAM rats responded similarly to RO pretreatment. A separate analysis for SHAM (*F*_1,52_ = 1.37, *p* = 0.25) and OBX (*F*_1,64_ = 2.87, p = 0.09) rats indicated no significant effect of RO on the cocaine-induced reinstatement (Fig. 6b).

Repeated administration of vehicle or MIR did not change behavioral responses during reinstatement of seeking behavior, represented as the increase of lever presses in experimental groups (a mixed ANOVA for the pretreatment × surgical condition × reinstatement (cocaine) × lever interaction: *F*_1,144_ = 0.06, *p* = 0.80; Fig. 6c). Further, an individual analysis revealed that the cocaine-induced reinstatement was similar in both groups (OBX: *F*_1,76_ = 0.12, *p* = 0.73; SHAM: *F*_1,68_ = 0.57, *p* = 0.45).

### The brain expression of the 5-HT_2C_ receptor

**Fig. 7:**
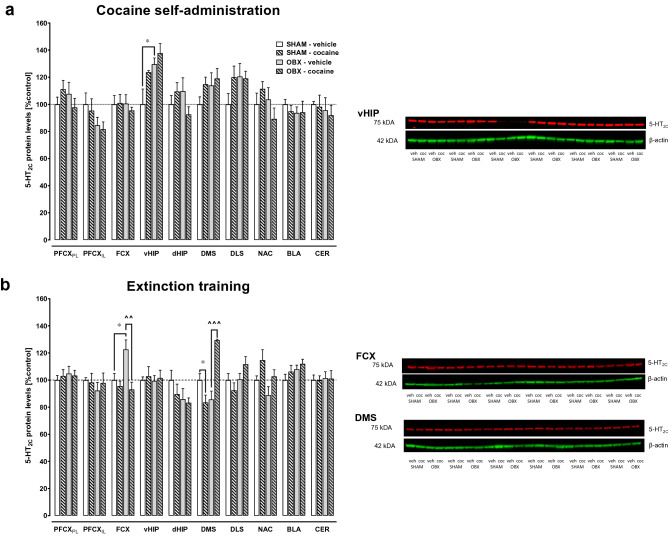
Changes in the expression of 5-HT_2C_ receptors in brain structures following cocaine self-administration (panel **a**, left) and following extinction training (panel **b**, left) in bulbectomized (OBX) and SHAM-operated rats. For comparison saline yoked administration is shown. Whole membranes showing 5-HT_2C_ receptor expression are presented (panels **a** and **b,** right). PFCX_IL_—infralimbic prefrontal cortex, PFCX_PL_—prelimbic prefrontal cortex, FCX—frontal cortex, vHIP—ventral hippocampus, dHIP—dorsal hippocampus, DLM—dorsolateral striatum, DMS—dorsomedial striatum, NAC—nucleus accumbens, BLA—basolateral amygdala, CER—cerebellum, veh—vehicle, coc—cocaine. All data are expressed as mean ± SEM from 6 rats/group and presented as % of control. The statistical analyses were done with Student *t* test. *p < 0.05 versus SHAM-vehicle (control), **^^***p* < 0.01, **^^^***p* < 0.001 versus OBX-vehicle

Cocaine self-administration per se did not change the 5-HT_2C_ receptor expression in examined brain structures in OBX and SHAM rats as compared to saline SHAM animals (Fig. 7a; Table 1). Removal of the olfactory bulbs induced an increase in the 5-HT_2C_ receptor expression only in the ventral hippocampus in yoked saline SHAM rats (*t*_10_ = − 2.43 *p* < 0.05; Fig. 7a).

**Table 1:** Results from two-way ANOVA on 5-HT_2C_ receptor expression in the several brain structures in bulbectomized and SHAM rats following cocaine self-administration and yoked saline delivery

Brain structure	PFCX_PL_	PFCX_IL_	FCX	vHIP	dHIP	DLS	DMS	NAC	BLA	CER
*F*_1, 20_	0.01	2.40	0.28	1.20	3.34	1.82	0.47	0.11	0.61	0.90
*P*	0.91	0.14	0.60	0.28	0.08	0.19	0.49	0.11	0.61	0.90

PFCX_IL_, infralimbic prefrontal cortex; PFCX_PL_, prelimbic prefrontal cortex; FCX, frontal cortex; vHIP, ventral hippocampus; dHIP, dorsal hippocampus; DLS, dorsolateral striatum; DMS, dorsomedial striatum; NAC, nucleus accumbens; BLA, basolateral amygdala; CER, cerebellum

A two-way ANOVA showed significant changes in the 5-HT_2C_ receptor expression in SHAM and OBX groups after 10-days extinction in rats self-administering cocaine or the vehicle group in the frontal cortex (*F*_1,20_ = 5.42, *p* < 0.05) and dorsomedial striatum (*F*_1,20_ = 39.88, *p* < 0.001). In the OBX vehicle group, an increase in the 5-HT_2C_ receptor expression was shown in the frontal cortex (*t*_10_ = − 2.64, *p* < 0.05), as compared to the SHAM vehicle group. In OBX cocaine group, there was a decrease in the 5-HT_2C_ receptor expression in the frontal cortex (*t*_10_ = 3.35, *p* < 0.01) and an increase in the dorsomedial striatum (*t*_10_ = − 7.01, *p* < 0.001), compared to OBX vehicle group (Fig. 7b).

## Discussion

In this paper, using rat models, we studied the association between voluntary cocaine intake and preexisting depression-like behavior, highlighting the potential mediating role of 5-HT_2C_ receptors in this comorbidity via regulating behavioral effects in vivo and changes in the signaling ex vivo.

Here, the behavioral investigation revealed for the first time that acute administration of RO—significantly and WAY—non-significantly (a reduction in active lever pressing and cocaine infusions by 34% and 46%, respectively) attenuated the reinforcing properties of self-administered cocaine in OBX rats. At the same time, MIR did not change this behavioral response in OBX rats. The same inhibitory effects of RO and WAY were observed in SHAM animals, and they reflect previous observations regarding RO [31], WAY [40], and lorcaserin, another 5-HT_2C_ receptor-preferring agonist [62]. Furthermore, the present findings support former observations in rhesus monkeys in which lorcaserin attenuated the discriminative stimulus effects of cocaine after acute administration and the reinforcing effects of cocaine after acute and repeated administration [63]. In contrast to the above studies, in male rhesus monkeys, acute or repeated lorcaserin treatment was ineffective toward the reinforcing properties of cocaine. It evoked nonselective decreases in operant responding maintained by a broad range of reinforcers [64]. The behavioral selectivity of 5-HT_2C_ drugs to decrease cocaine self-administration/cocaine choice is related to the unit cocaine dose. In other words, 5-HT_2C_ drugs show greater specificity and selectivity at lower cocaine doses (here their effects studied with 0.5 mg/kg/infusion cocaine self-administration).

In our study, the similarity of behavioral results for OBX and SHAM groups following the administration of 5-HT_2C_ receptor agonists suggests that the reinforcing activity of cocaine is under control of these receptors, despite the presence of a depression-like phenotype. Moreover, our ex vivo analyses also showed that cocaine self-administration raised the expression of the 5-HT_2C_ receptor protein in the ventral hippocampus in OBX and SHAM animals, which indicates that this receptor within the investigated brain area acts as a molecular target controlling cocaine reinforcement. This finding extends a previous observation with local (intra-ventral tegmental area, intra-amygdala, or intra-medial prefrontal cortex (mPFCX)) infusions of 5-HT_2C_ receptor agonists that reduce the above cocaine behavior [32, 64, 65]. On the other hand, tonic activation of 5-HT_2C_ receptors does not alter the reinforcing effects of cocaine, as MIR altered neither the number of active lever pressing nor the number of cocaine infusions. This discovery extends our previous observations with the 5-HT_2C_ preferring-receptor antagonist SDZ SER-082 which also did not change cocaine reinforcement in rats [67].

In the next set of experiments, we found that acute pretreatment with 5-HT_2C_ receptor agonists or MIR administration reduced cocaine-seeking behavior evoked by cue- or cocaine priming in SHAM and OBX animals. Such inhibitory effects of the above drugs speak for their effective prevention effects for cocaine use intake and relapse*.* This report, for the first time, indicated the efficacy of RO and WAY in effectively inhibiting the recurrence of cocaine-seeking behavior in both phenotypes of rats. That means that 5-HT_2C_ receptor agonism demonstrates the potential clinical utility to reduce cocaine-seeking enhanced by co-existing depression. It should underline that 5-HT_2C_ receptor agonism is linked to sedation and reduction in motor behaviors; however, such nonspecific impairment related to inhibition of relapse was not likely to depend on RO or WAY. RO doses used in this study, were much lower than those which weakened motor functions in the rotarod test [31]. Further, both 5-HT_2C_ receptor agonists had no significant effect on the number of inactive lever presses during the reinstatement of seeking behavior, which indicates the specificity of their action (see also previous findings by [31, 67]). At the end, the anti-relapse effects of RO and WAY were blocked by 5-HT_2C_ receptor antagonists, which proves that the recurrence of cocaine-seeking behavior depends on the stimulation of these receptors [31, 38]. Our present study on the 5-HT_2C_ receptor stimulation in SHAM rats supports previous observations [31, 35, 38]. As indicated in more recent studies, selective 5-HT_2C_ receptor activation decreased drug-seeking behavior in non-human primate models of cocaine abuse [42, 68, 69]. In a separate randomized controlled trial, lorcaserin delayed intravenous choices and decreased craving under some conditions in regular cocaine users; however, some positive subjective effects of cocaine were enhanced by lorcaserin [71].

Since 5-HT_2C_ receptor antagonists per se did not reduce cocaine-seeking behaviors [38, 66], the inhibitory response of MIR seen in this appearance is of special interest. This drug, apart from 5-HT_2C_ receptors, also targets 5-HT_2A_ and 5-HT_3_ receptors [72], which are engaged in the control of cocaine-seeking behaviors [65, 66, 72–74]. MIR also enhances noradrenergic neurotransmission via antagonism of the central α2-adrenergic autoreceptors and heteroreceptors [72], however, their role in cocaine relapse has not been investigated so far and it is recommended for future research. Hence, 5-HT_2C_ receptor agonists, by eliminating animals’ behavioral responses to the contingent cue, become important modulators of subsequent behavior.

Our findings also demonstrate that repeated administration of 5-HT_2C_ receptor agonists—RO and WAY—during extinction training resulted in significant inhibition of the reinstatement of cocaine-seeking behavior induced by the presentation of the conditioned stimulus. In contrast, a weaker (non-significant) reduction in the reinstatement after administration of a priming dose of cocaine in SHAM and OBX groups, was observed. The different effects of chronic administration of 5-HT_2C_ receptor agonists during extinction training on the reinstatement of seeking behavior induced by different stimuli (cue vs. cocaine) may be due to the involvement of, among others, localization of these receptors to separate regions of rat (sub)brain. For example, the prelimbic prefrontal cortex and infralimbic cortex, as well as the lateral orbitofrontal cortex and medial part of the orbitofrontal cortex, play an opposite role the reinstatement of cocaine-seeking [75–78] and in the localization of 5-HT_2C_ receptor mRNA and protein in those areas [70, 79]. Furthermore, a lesion of the medial part of the orbitofrontal cortex enhances the reinstatement of seeking behavior induced by the priming dose of cocaine [76]. In contrast, a pharmacological inactivation or lesion of the lateral orbitofrontal cortex inhibited exploratory seeking behavior for cocaine after the presentation of the conditional stimulus [75, 76, 78].

Secondly, a pharmacological analysis with drug microinjections demonstrated that stimulation of the 5-HT_2C_ receptor in the prelimbic and infralimbic subregions (but not in the anterior cingulate cortex) of the mPFCX or the central amygdala (but not in the basolateral amygdala) suppressed reinstatement of cocaine-seeking behavior, an effect reversed by co-infusion of a selective 5-HT_2C_ receptor antagonist [64, 65]. Thirdly, only one report shows variation in the 5-HT_2C_ receptor expression in different brain areas during high cocaine cue reactivity in humans and animals [46]. The latter authors indicated that enhancement in drug-associated lever presses reinforced by the discrete cue complex was correlated with lower 5-HT_2C_ receptor protein expression in the mPFCX and blunted sensitivity to the suppressive effects of WAY, a selective 5-HT_2C_ receptor agonist [46]. In this context, our present neurochemical analyses revealed that cocaine abstinence with extinction training only evoked a significant decrease in the dorsomedial striatum. To check whether this change contributes to cue- or cocaine-related drug seeking, further pre- and post-training microinjections of 5-HT_2C_ agonists are needed.

As shown above, MIR given acutely reduced cocaine-seeking, but its repeated administration—at a dose effectively blocking cocaine actions—during extinction failed to inhibit cue or the reinstatement of cocaine-seeking behavior. This finding is in direct contrast with previous observations, the daily dosing of MIR (30 mg/kg, *ip*) for 30 days significantly attenuated the induction of cocaine-induced locomotor sensitization [80–82]. Additionally it attenuated the re-acquisition of cocaine-seeking responses in rats [80, 83]. It should be stressed that chronic dosing of MIR (30 mg/kg, *ip*) during cocaine withdrawal reduced depression- and anxiety-like behaviors that characterize cocaine withdrawal in rats [85]. Since in those and the present studies, observations at different periods of cocaine abstinence, different times of drug administration, as well as MIR doses were applied, it could justify the occurrence of differences.

The development of a depressive status by removing the olfactory bulbs produced significant and time-dependent increases in 5-HT_2C_ receptor levels in the rat ventral hippocampus and frontal cortex, as compared to SHAM saline controls. The increased protein expression of these receptors is likely to be responsible for the intensification of primary depressive disorders in OBX rats and thus may lead to an increase in the reinstatement of cocaine-seeking behavior observed in these animals in our previous studies [52–54]. Furthermore, significant enhancement of 5-HT_2C_ receptors localized in the dorsomedial striatum was reported in animals co-expressing depression and CUD. The latter brain area mediates the acquisition and performance of cocaine-seeking behavior [86] and pharmacological activation of the dorsomedial striatal 5-HT_2C_ receptors leads to a reduction in dopaminergic neurotransmission [86, 87], while a local reduction in the expression of the 5-HT_2C_ receptor protein level mediates the restoration of normal dopaminergic transmission [89]. According to previous observations, an increase in 5-HT_2C_ receptor protein expression in the dorsomedial striatum in laboratory animals was associated with anxiety [11, 89, 90] and depressive disorders [17, 91, 92].

The present study not fully explained the role of the 5-HT_2C_ receptor in comorbid depression and CUD. As shown in Introduction both 5-HT_2C_ receptor agonists, inverse agonists, and antagonists exhibit antidepressant-like activity in animal models of depression regulated monoamine levels in brain structures related to depression [11, 20, 21, 23–27, 72, 93]. Nevertheless, our research backs the further development of pharmacological strategies with drugs targeting the 5-HT_2C_ receptor for treating comorbid depression and CUD, seeing that treatments based on manipulations of 5-HT_2C_ receptors inhibit the rewarding properties of cocaine as well as the reinstatement of seeking behavior induced by conditional and unconditional stimuli.

## Supplementary Information

Below is the link to the electronic supplementary material.

Supplementary file 1 (DOCX 2465 KB)

## Data Availability

The data used in the present study are available from the corresponding author on reasonable request.

## Abbreviations

5-HT: Serotonin
5-HT_2C_: Serotonin 2C receptor
CUD: Cocaine use disorder
FR: Fixed ratio
MIR: Mirtazapine
mPFCX: Medial prefrontal cortex
OBX: Olfactory bulbectomy
RO: RO 60-0175
WAY: WAY 161503
