# Supplementary material for: Evaluation of the 5-HT2C receptor drugs RO 60-0175, WAY 161503 and mirtazepine in a preclinical model of comorbidity of depression and cocaine addiction
Source: Pharmacol Rep. 2022 Nov 14;75(1):99–118. doi: 10.1007/s43440-022-00428-2 (PMC9889480; doi:10.1007/s43440-022-00428-2)

**Supplement 1**

**Figure S1.** Corresponding membranes from Western blot analyses of 5-HT_2C_ receptor and loading controls (β-actin) in brain structures following cocaine self-administration in bulbectomized (OBX) and SHAM-operated rats. Veh – yoked saline, coc – cocaine self-administration, **A**: PFCX_IL -_ infralimbic prefrontal cortex, PFCX_PL -_ prelimbic prefrontal cortex, FCX - frontal cortex, vHIP - ventral hippocampus, dHIP - dorsal hippocampus, **B:** DLS - dorsolateral striatum, DMS - dorsomedial striatum, NAC - nucleus accumbens*,* BLA - basolateral amygdala*,* CER - cerebellum.

**A.**

*
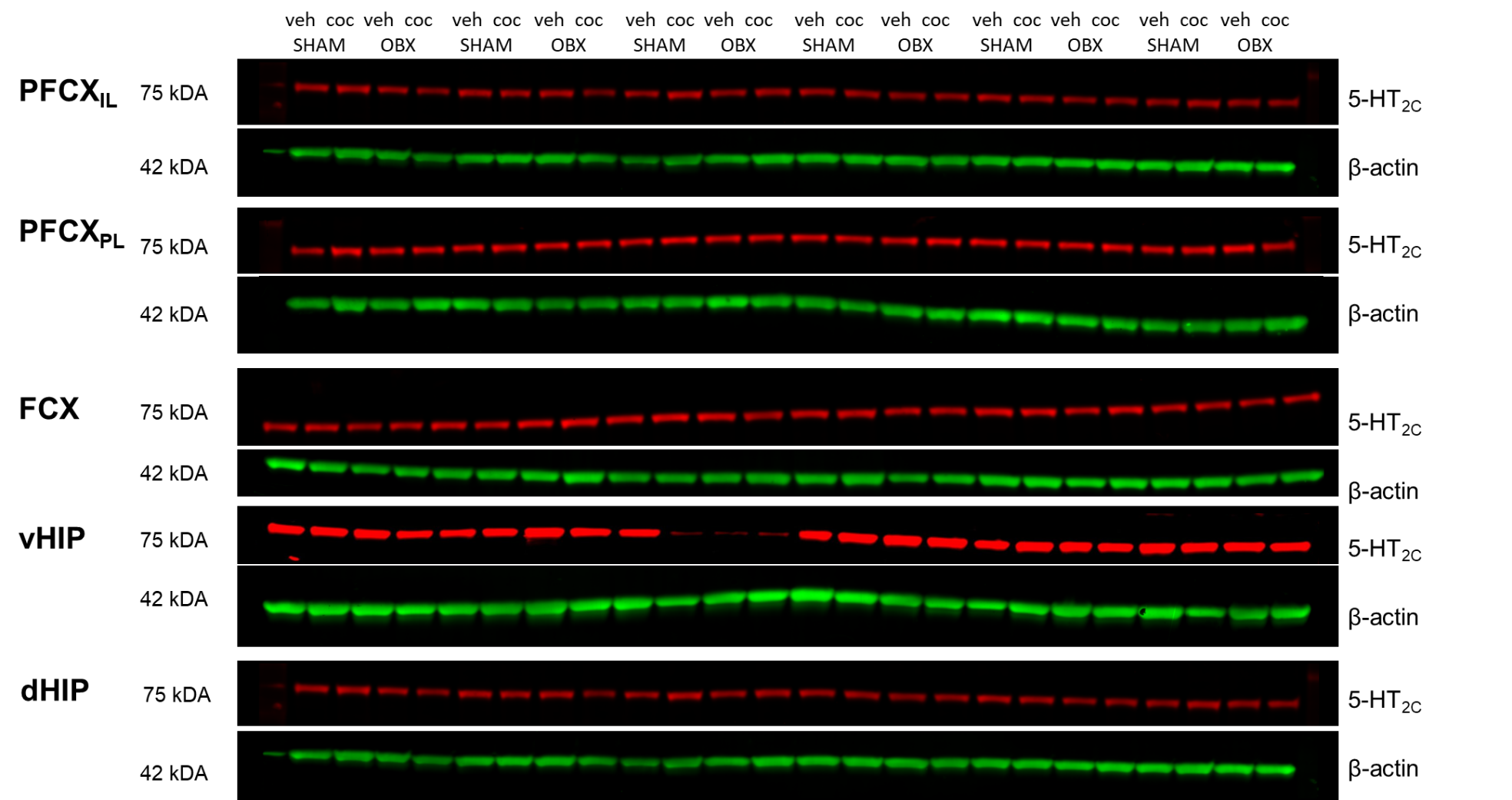
*

**B.**

*
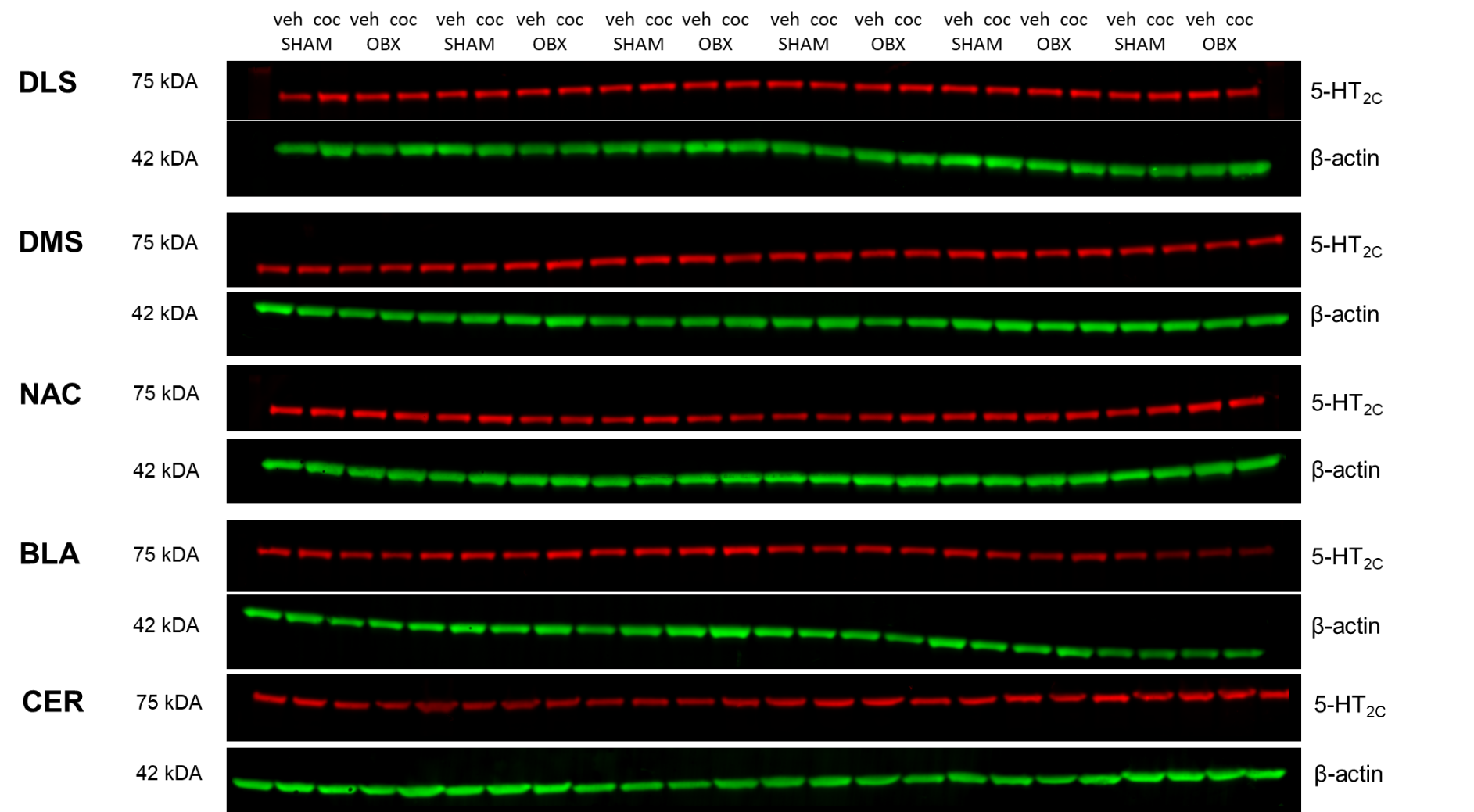
*

**Figure S2.** Corresponding membranes from Western blot analyses of 5-HT_2C_ receptor and loading controls (β-actin) in brain structures following cocaine self-administration and extinction training in bulbectomized (OBX) and SHAM-operated rats. Veh – yoked saline, coc – cocaine self-administration, **A**: PFCX_IL -_ infralimbic prefrontal cortex, PFCX_PL -_ prelimbic prefrontal cortex, FCX - frontal cortex, vHIP - ventral hippocampus, dHIP - dorsal hippocampus, **B:** DLS - dorsolateral striatum, DMS - dorsomedial striatum, NAC - nucleus accumbens*,* BLA - basolateral amygdala*,* CER - cerebellum.

**A.**

**
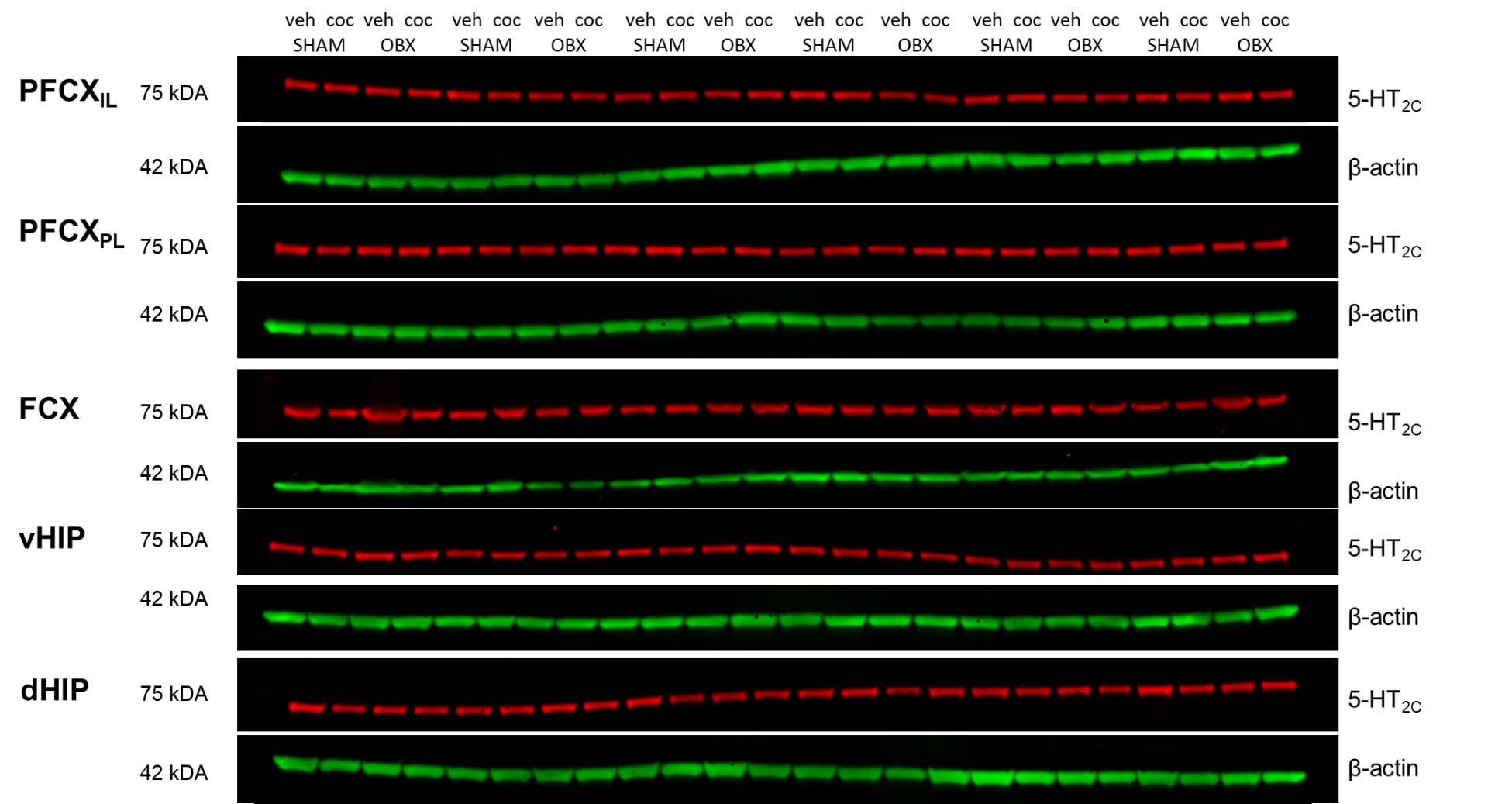
**

**B.**


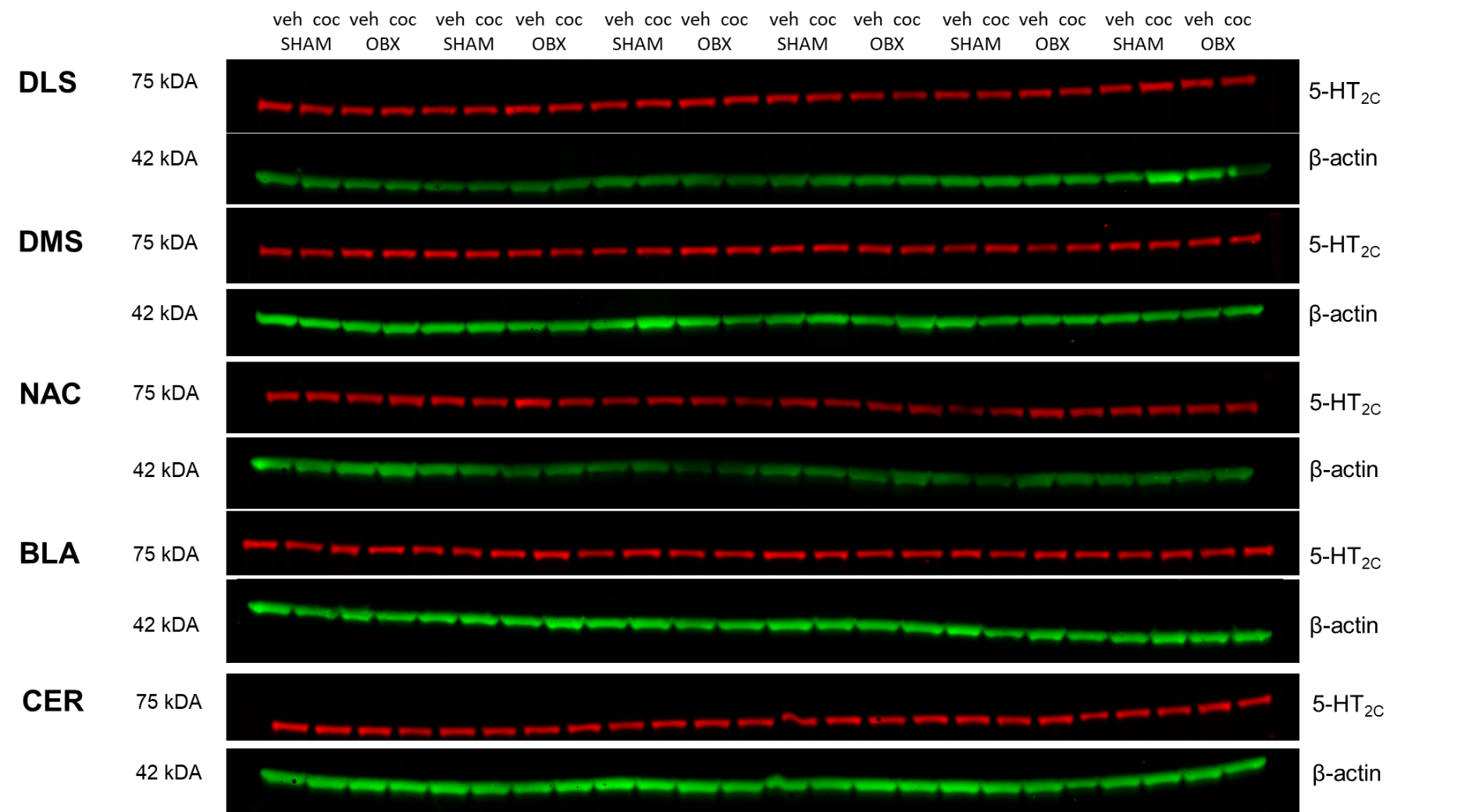

Supplement: Supplementary file 1 — (DOCX 2465 KB) [file 43440_2022_428_MOESM1_ESM.docx]
